# Effective Route Maintenance and Restoration Schemes in Mobile *Ad Hoc* Networks

**DOI:** 10.3390/s100100808

**Published:** 2010-01-21

**Authors:** Byung-Seok Kang, In-Young Ko

**Affiliations:** Department of Computer Science, Korea Advanced Institute of Science and Technology (KAIST), 335 Gwahangno, Yuseong-gu, Daejeon, 305-701, Korea; E-Mail: iko@kaist.ac.kr

**Keywords:** route maintenance, route restoration, mobile *ad hoc* network

## Abstract

This study proposes a location-based hybrid routing protocol to improve data packet delivery and to reduce control message overhead in mobile *ad hoc* networks. In mobile environments, where nodes move continuously at a high speed, it is generally difficult to maintain and restore route paths. Therefore, this study suggests a new flooding mechanism to control route paths. The essence of the proposed scheme is its effective tracking of the destination’s location based on the beacon messages of the main route nodes. Through experiments based on an NS-2 simulator, the proposed scheme shows improvements in the data packet delivery ratio and reduces the amount of routing control message overhead compared with existing routing protocols such as AODV, LAR, ZRP and AODV-DFR.

## 1. Introduction

A mobile *ad hoc* network (MANET) is a self-configuring network of mobile devices connected by wireless links. In a MANET, each node acts as a router to establish end-to-end connections, and because the network topology between sources and destinations frequently changes, it is difficult to maintain and restore a route. To deal with these issues, several routing protocols for MANETs have been proposed. However, these protocols are simulated with low numbers of network nodes with low node mobility. Moreover, to track mobile destination, they require large a number of control messages.

This study suggests a location-based hybrid routing protocol for a large number of high-mobility nodes. The proposed routing scheme effectively manages the maintenance and restoration of a route. The basic operation of the scheme is similar to the *Ad Hoc* On-demand Distance Vector (AODV) algorithm [1], as the suggested scheme adds a greedy forwarding algorithm, the location information of a Global Positioning System (GPS), and directional forwarding [2] to the AODV algorithm. In addition, the proposed algorithm can reduce routing overhead. The main route nodes broadcast beacon messages, which have the location of the destination, to their one-hop neighbors. This approach provides efficient tracking of a mobile destination and reduces messages for routing.

To evaluate the performance of the proposed scheme, the data packet delivery ratio and routing control overhead messages are analyzed and compared with existing routing protocols of AODV, Location-Aided Routing (LAR) [3], Zone Routing Protocol (ZRP) [4], and AODV-DFR [5].

The remainder of this paper is organized as follows: Section 2 reviews the previously known MANET and related algorithms. Section 3 develops the proposed routing scheme. In Section 4, the proposal is verified by simulation. Finally, Section 5 summarizes the contributions of this study.

## 2. Related Works

In a MANET, routing protocols can be divided into two major categories - proactive routing and reactive routing. Proactive routing protocols, also known as table-driven routing protocols, contain information on every node and update the routing table information periodically. In DSDV [6], OLSR [7], and WRP [8], typical proactive routing protocols, the source node is equipped beforehand with information appertaining to the pathway of the destination node before it send data packets there. As a fatal drawback of the proactive routing approach, however, a mass of route-updating messages flood the entire network periodically to maintain the route information fresh. Furthermore, each node unnecessarily stores the full set of route information, especially in a highly mobile environment where the routing table of a node is updated frequently for dynamic topology. Each node must find the latest broadcast routing path information periodically. Such periodic updates cause unnecessary network overhead.

Reactive routing protocols, also known as on-demand routing protocols, do not conserve the routing table information; instead, they execute a route discovery procedure to determine a route to the destination only when the source node requires a path to the destination node. Once a route has been discovered, the route is maintained until the destination becomes inaccessible or the route is no longer desired. AODV, DSR [9] and the Temporally Ordered Routing Algorithm (TORA) [10] are representative examples of reactive protocols. Particularly with a large number of nodes, reactive routing protocols are more appropriate than a proactive routing approach.

In the following subsections, existing routing protocols that aim to improve the data packet delivery ratio and reduce routing overhead are explained.

### 2.1. Algorithms to improve the data packet delivery ratio

Hybrid routing strategies, which draw upon the most advantageous features of both proactive and reactive mechanisms, are designed to improve the communication quality for active routes with lower overhead. As a hybrid routing strategy, ZRP has been proposed to provide a hybrid routing framework for a locally proactive and globally reactive approach so as to minimize the sum of the proactive and reactive control overhead. For the zone radius, the main idea of ZRP, each node proactively advertises its link state over a fixed number of hops. These local advertisements over nodes offer an updated view of the routing zone collection of all nodes (or links) that are reachable within the zone radius. The nodes, termed peripheral nodes, are located on the boundary of the routing zone and play an important intermediate role in reactive zone-based route discovery.

The other means of improving the data packet delivery ratio is via a Clustering algorithm [11–13]. Cluster head-Gateway Switch Routing (CGSR) is a typical cluster-based hierarchical routing method. A stable clustering algorithm, Least Cluster head Change (LCC), is used to partition the entire network into several clusters with the selection of a cluster head for each cluster. A mobile node that belongs to two or more clusters is a gateway connecting them, and data packets are routed through paths with the format of a “Cluster head-Gateway” between every source and destination pair.

Integrated Services (IntServ) [14] and Differentiated Services (DiffServ) [15] also guarantee a good quality of packet delivery ratio. An IntServ protocol such as the Bandwidth Reservation over *Ad Hoc* Wireless Networks (BRAWN) [16] mechanism not only guarantees certain QoS levels but also naturally distributes the traffic more evenly among network nodes, similar to load balancing through a rich end-to-end QoS solution mechanism. On the other hand, DiffServ such as a Stateless Wireless *Ad Hoc* Network (SWAN) [17] can be used to classify network traffic into different priority levels and apply priority scheduling and queuing management mechanisms to obtain QoS guarantees.

### 2.2. Algorithms to reduce routing overhead

Compared to the proactive approach, the reactive approach is high-powered at a lower network overhead and has less data transmission delay and no periodical routing update. The LAR protocol has been presented as an On-Demand protocol based on source routing. The protocol utilizes location information to limit the area when seeking to discover a new route to a smaller “request zone”. As a consequence, the number of route request messages is reduced.

The Greedy Perimeter Stateless Routing (GPSR) protocol [18], another Geo-routing protocol, holds only neighbor location information for delivering data packets. This approach is appropriate for dense wireless networks due to its small number of per-node routing states and low routing message complexity.

The Fisheye technique, another algorithm to reduce routing message overhead, is used for Fisheye State Routing (FSR) [19] and AODV-DFR. First, FSR exchanges all of the link state information with closer neighbors instead of flooding the information over the network. Regarding link state table management, FSR uses an up-to-date technique. Secondly, in FSR, the link state information is swapped periodically instead of being event-triggered as a method to prevent frequent link state updates which cause link breaks and unreliable wireless links. Moreover, periodic broadcasts of the link state information are operated using different frequencies on different entries that depend on their hop distances to the current node.

Finally, protocols similar to the improved original AODV routing protocol were presented in other studies [20,21]. In these two protocols, the originating node can estimate the distance to the destination node and set the initial value of the TTL field in the RREQ message based on that estimated value. This is done using a simple modification of TTL field technique, which may result in an unnecessarily large number of control packets traveling through the network.

## 3. Proposed Algorithm

As explained in the previous section, many studies that sought to improve the data packet delivery ratio have been done, but these works leave a considerable number of routing overhead messages. This section describes a detailed scheme to improve the data packet delivery ratio while reducing the number of routing overhead messages via one-hop neighbor beaconing of the main route nodes.

### 3.1. Directional forwarding

**Figure 1.**
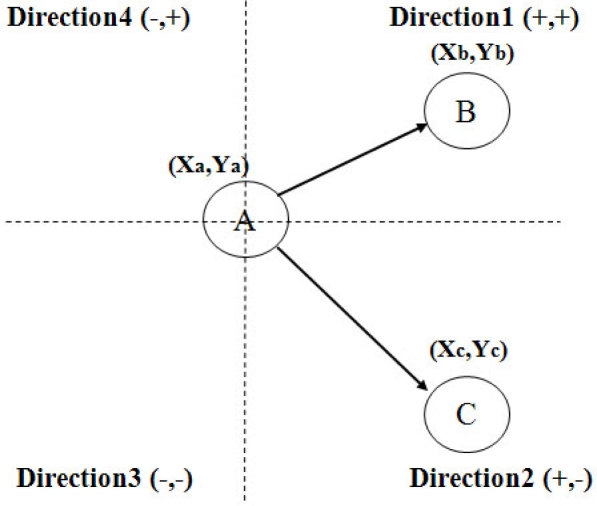
Four-way directional forwarding.

Figure 1 shows the proposed directional forwarding algorithm. Each node recognizes its location through GPS. A one-hop neighbor node, which is at a distance of one hop from the main route, recognizes the location of the main route node through a beacon message. In the figure, node A contains information about its location (Xa, Ya). In this situation, node A broadcasts its location information using a beacon message. Nodes B and C store the location of node A in each routing table. Four-directional forwarding, directions 1 to 4, is then applied. Direction 1 is (+, +), Direction 2 is (+, –), Direction 3 is (–, –), and Direction 4 is (–, +). In Figure 1, Node B locates direction 1 from node A, and node A sets the position of direction 3 from node B. In the same manner, node C locates direction 2 from node A and node A locates direction 4 from node C. Though two-, four-, eight-, and 16-way directions can be applied, four-way direction shows the best performance with the least routing overhead. Using two-way direction, it causes many routing overhead as similar data packet delivery ratio. Furthermore, using eight- and 16-way direction, they result very low data packet delivery ratio because there are not enough control messages to trace the destination’s location.

### 3.2. Basic operation of the algorithm

**Figure 2.**
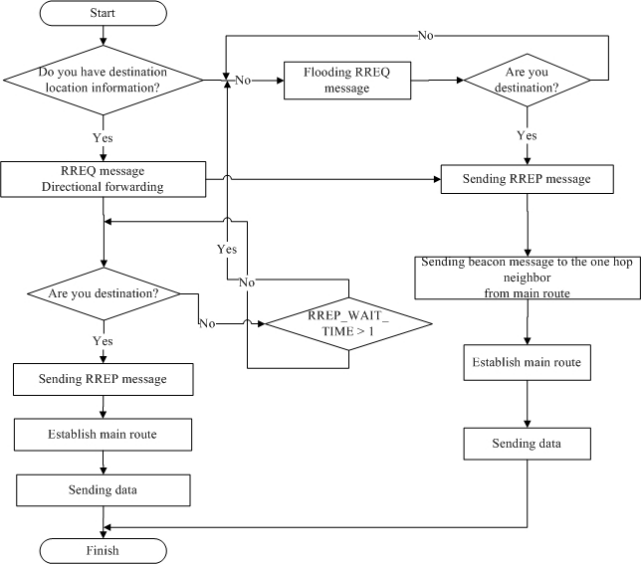
Basic operational flow chart.

The proposed algorithm uses four message types: beacon, Route Request (RREQ), Route Reply (RREP), and Route Error (RERR). Originally, any node can broadcast beacon messages but in the proposed scheme, only the main route nodes send beacon message to one-hop neighbor nodes by setting the TTL value of the beacon message to 1. RREQ and RREP are used in the route discovery process. RERR is used in the route maintenance and restoration process. Figure 2 depicts the basic operations of the proposed algorithm.

#### 3.2.1. Local routing table

As reactive routing protocols typically operate based on a routing table, the proposed protocol also has a local routing table. The local routing table is updated whenever it receives RREQ, RREP, and RERR messages. Table 1 shows the proposed local routing table, which has more fields than the AODV routing table. The function of each field is described below. The DestID field stores the destination node ID using direction values that range from 1 to 4. The distance field stores the distance value between two neighbor nodes, from 1 to 250, as the transmission range of the node. The HopCnt field indicates the hop count information to the destination node. SeqNo is given automatic sequential numbers, which specify a larger sequence number as newer route information. Lifetime is the lifetime of the route information; the information with a higher lifetime value remains in the routing table longer. Finally, the state field determines whether or not the route information is valid. If the lifetime field is 0 or if the state field reads INVALID, the routing tab entry is deleted.

#### 3.2.2. Route maintenance and restoration

Route maintenance and restoration are important factors in MANETs. In this subsection, the proposed route maintenance and restoration schemes are discussed.

**Figure 3.**
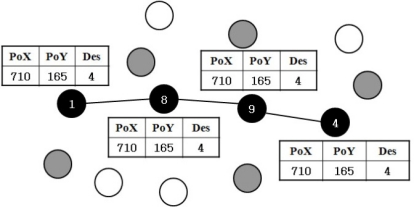
Route maintenance.

Figure 3 illustrates the route maintenance method between the source node and the destination node. The black nodes are the main route nodes, the gray nodes are one-hop distance nodes from the main route node, and white nodes are two hops or more from the main route nodes. For instance, if a white node moves to a one-hop distance from the main nodes, it will then change its status to a gray node. Under the same approach, if a gray node moves away from the main route node by more than two hops, it will change its status to a white node. The difference between gray and white nodes is whether or not the node receives a beacon message from the one of the main route nodes.

In Figure 3, node 1 transmits the information of the location of the destination node to its one-hop neighbor node 8. This information is broadcast with a beacon message every second to its one-hop neighbor nodes by setting the TTL of beacon message to 1. Similarly, nodes 8, 9, and 4 broadcast beacon messages to their neighbors that have the location information and address of the destination node. Next, the one-hop-distant neighbor nodes from the main route nodes write the routing information on each routing table. The purpose of beaconing is to find the location of the destination immediately when route failure occurs along the main route nodes. This procedure is used as an effective type of local repair.

Regarding the local repair procedure of an AODV, it broadcasts an RREQ message to find the destination node set as TTL field = 2 when a node finds a link failure. If no destination node is found, it then sends an RERR message back to all active nodes that are dependent on the broken link. When every node receives the RERR message, those nodes delete the route of the broken link. Particularly for a source that receives such an RERR message, it can restart the route discovery procedure; a stale route is expired according to a timer-based technique in what is known as an AODV local repair procedure.

**Figure 4.**
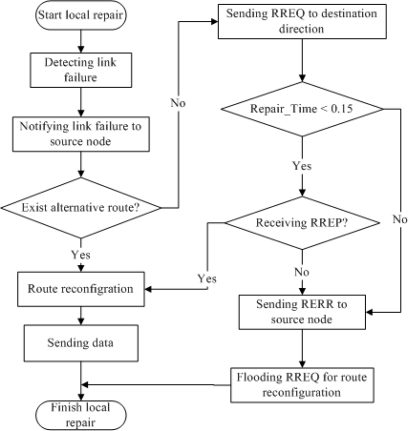
Local repair flow chart.

In the present scheme, when an upstream node detects a link failure, the node sends an RREQ message toward only the direction of the destination. These procedures contribute toward reducing the routing message overhead. Figure 4 shows a flow chart of the proposed local repair procedure.

### 3.3. Packet forwarding

Once a source node recognizes the location of the destination, the source node selects one node from the routing table toward the destination direction. If many candidate nodes are selected, the source node selects the node of the minimum hop count and the highest sequence number. Nevertheless, if it still has two or more candidate nodes, it finally selects the one node with the longest distance value. The distance is determined from the distance field value of the routing table. If there is no suitable node for packet forwarding, local repair procedures are executed.

**Figure 5.**
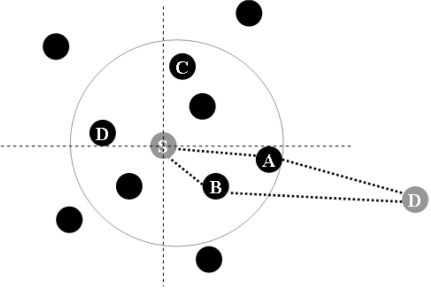
Packet forwarding.

Figure 5 depicts source node S selecting one of the forwarding packets among nodes which locate in its transmission range. First, node S selects A and B nodes having the same direction (direction = 2) to the destination from its routing table. Subsequently, if they have the same hop count value and sequence number, node S then compares the distance field value of each one. As described in Figure 5, node A is finally selected as the packet forwarding node that has a larger distance value than node B.

### 3.4. Control routing overhead

*Ad Hoc* On-demand Distance Vector with Controlled Flooding (AODV-CF) [22] uses two new messages to determine an alternative route in order to prepare for link failure. The first is a Controlled message, and the second is a Controlled-ACK message. Once the alternative node is set, there is no periodical update until a link failure occurs. This can cause critical problems when nodes are moving fast. In such an environment, the alternative nodes no longer perform their function as alternative nodes. In this circumstance, in the suggested scheme the main nodes send a beacon message containing information pertaining to the location of the destination to their one-hop neighbor nodes periodically every second.

**Figure 6.**
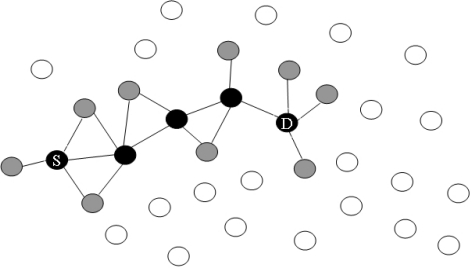
Limitation in sending beacon messages.

The black node is the main route node described in Figure 6. The gray node, the one-hop neighbor from the main node, receives information regarding the location of the destination as periodically transmitted from the main route nodes. The main route nodes continue to send location information to their new one-hop neighbors unless gray nodes move far away from the main route nodes. This mechanism works effectively with a large network. In existing hybrid algorithms, every node sends a beacon to its one-hop neighbor, which creates a tremendous amount of routing message overhead. Although the proposed scheme has some routing message overhead, it has small number of messages compared to the previous hybrid routing protocols.

## 4. Simulation Analysis

In this section, the experimental results of the proposed routing algorithm as evaluated with the NS-2 simulator are explained. The simulation results prove that the proposed scheme outperforms traditional routing protocols.

### 4.1. Network model and performance metrics

The superiority of the proposed algorithm was verified with the environment settings shown in Table 2. In particular, simulations were run for 200 simulated seconds in a 10 km × 10 km large rectangular network space.

Three metrics were used to evaluate the performance: the percentage of data packets that were received at the destination node, the control messages overhead, and the average number of control messages being used to deliver a message to a destination. The data packet delivery ratio refers to the percentage of the number of packets that are received at the destination to the number of packet sent by the source. The larger this metric is, the more efficient the MANET will be. The control message overhead metric represents the total number of control message packets per second in the experiment network. By combining the two metrics, the average number of control messages being used to deliver a message to a destination, can be calculated and finally used to present the performance of each algorithm. The simulation results of the proposed scheme are compared with the results of AODV, LAR, ZRP and AODV-DFR.

### 4.2. Data packet delivery ratio

The simulation results describe the data packet delivery ratios of the AODV, LAR, ZRP, AODV-DFR schemes and the proposed routing algorithm. In the simulations, 5 m/s and 20 m/s node movements are considered.

**Figure 7.**
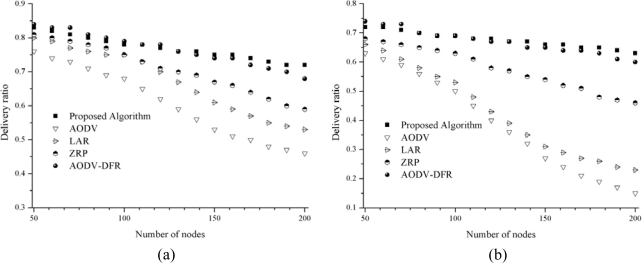
Data packet delivery ratio. (a) 5 m/s node movement; (b) 20 m/s node movement.

In Figure 7(a), as the node count increases, the packet delivery ratio is decreased. When 50 nodes exist in the network, all protocols show a data packet delivery ratio greater than 75%. They show different success ratios as the number of nodes increases. However, the proposed scheme results in a data packet delivery ratio of 72% even in case of 200 nodes used because each node efficiently stores the node information of its surrounding neighbors. In contrast, the performance degradations in AODV and LAR are significant as the number of nodes increases. This results because there are frequent route changes and reconfigurations in a densely populated environment. When the number of nodes is small, AODV-DFR, LAR and ZRP work well and provide high delivery ratios, while the delivery ratio of AODV quickly falls to less than 70%. The AODV routing protocol is not efficient when numerous nodes exist in a large network.

The performance levels of AODV, LAR, ZRP, AODV-DFR and the proposed routing protocols are also investigated at high speeds of 20 m/s (72 km/hour) and while varying the number of nodes. The increased speed of node movement stresses the routing protocols and introduces several new problems, as shown in Figure 7(b). As Figure 7(b) shows, every routing protocol experiences large-scale degradation compared to the 5 m/s node movement except for AODV-DFR and the proposed algorithm. At a node movement speed of 20 m/s, the data packet delivery ratios of the AODV and LAR falls to less than 30% with more than 150 nodes. The simulation results prove that AODV and LAR are not appropriate in high-mobility environments.

In both cases of different node movement speeds, even though the proposed algorithm results in a higher data packet delivery ratio, the AODV-DFR and proposed algorithms show similar delivery ratios. This occurs because AODV-DFR creates more routing control messages than the proposed protocol as the following subsection shows.

### 4.3. Control message overhead

**Figure 8.**
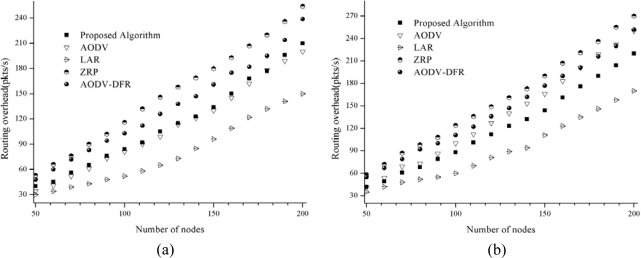
Control message overhead. (a) 5 m/s node movement; (b) 20 m/s node movement.

The simulation results of the control message overhead are presented in Figures 8(a,b). At a node speed of 5 m/s, the proposed scheme shows results that are better than AODV-DFR and ZRP and similar to AODV, whereas at a node speed of 20 m/s, the proposed scheme shows results that are better than those of AODV, AODV-DFR and ZRP. The proposed protocol uses slightly more control messages than LAR due to its use of an additive one-hop beacon message from the main route nodes.

### 4.4. Number of control messages per delivered message

**Figure 9.**
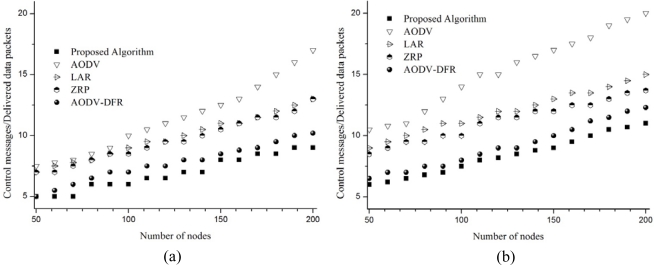
Number of control messages per delivered message. (a) 5 m/s node movement; (b) 20 m/s node movement.

The results of previous subsections 4.2 and 4.3 can be combined into a graph that represents number of control messages being used to deliver a message. In case of 5 m/s of node movement, as Figure 9(a) and Table 3 show, the proposed algorithm uses less control messages of 11.7% on average than AODV-DFR and 38.7%, 42.6%, and 65.1% than ZRP, LAR, and AODV respectively. And in case of 20 m/s of node movement, the proposed algorithm shows better result in reducing control messages of 9.5% on average compared with AODV-DFR and 33.9%, 43.4%, and 82.4% compared with ZRP, LAR, and AODV respectively as shown in Figure 9(b) and Table 3. These results clearly show that the suggested algorithm effectively reduces the number of control messages to deliver a message than other existing algorithms especially on the high speed of node movement.

## 5. Conclusions

Several routing protocols for MANETs have been studied recently. This research, including simulations of different routing protocols, is limited to illustrating the performance levels as evaluated using a relatively small number of nodes, leaving the manner of scaling the protocols to populations with larger nodes as a future line of research. As the number of users over wireless environments has increased, wireless protocols are also required to scale-up before they can fit increasingly larger populations of nodes.

In this study, an effective MANET routing protocol suitable for larger and faster node mobility network environments is presented. To establish the routing protocol, we applied directional forwarding with one-hop beaconing from the main route nodes for route maintenance and restoration, a routing algorithm operating based on GPS location information, and greedy packet forwarding.

The NS-2 simulator was used to evaluate the proposed protocol. The evaluations showed that the proposed scheme increases the data packet delivery ratio over that of industrialized MANET routing protocols. A reduction of the control message overhead in large and high-node mobility networks was also obtained.

## Figures and Tables

**Table 1.** Local routing table.

**DestID**	**Direction to Destination**	**Next Node**	**Distance**	**HopCnt**	**SeqNo**	**Lifetime**	**State**
Node 14	1	Node 17	220	4	341	10	VALID
Node 46	3	Node 54	160	5	268	0	INVALID
Node 75	4	Node 32	130	5	342	5	VALID

**Table 2.** Simulation environments.

**Environments**	**Value**
Simulation time	200 sec.
Area size	10 km × 10 km
Transmission range	250m
Movement speed	5 m/s, 20 m/s
Packet size	512 Bytes
Number of nodes	50∼200
Number of UDP connection	5∼20
MAC	IEEE 802.11 WLAN DCF
Data sending rate	4 kBps

**Table 3.** Average number of control messages per delivered message.

**Node speed**	**Proposed**	**AODV-DFR**	**ZRP**	**LAR**	**AODV**
5 m/s	6.94	7.75	9.63	9.89	11.46
20 m/s	8.39	9.19	11.23	12.03	15.3
